# Supplementary material for: Cardiolipin, and not monolysocardiolipin, preferentially binds to the interface of complexes III and IV
Source: Chem Sci. 2022 Oct 26;13(45):13489–98. doi: 10.1039/d2sc04072g (PMC9682889; doi:10.1039/d2sc04072g)
Supplement: SC-013-D2SC04072G-s008 [file SC-013-D2SC04072G-s008.pdf]

Electronic Supplementary Material (ESI) for Chemical Science.
This journal is © The Royal Society of Chemistry 2022

Cardiolipin, and not monolysocardiolipin, preferentially binds to the interface of Complexes III and IV

Robin A. Corey, Noah Harrison, Phillip J. Stansfeld, Mark S.P. Sansom, Anna Duncan

Supplemetary Data

1. Coordinates of CL and MLCL binding sites, as identified by PyLipID, for yeast, mouse and human supercomplexes (6 files)

2. Itp file for CL > MLCL used for FEP

Supplemetary Figures

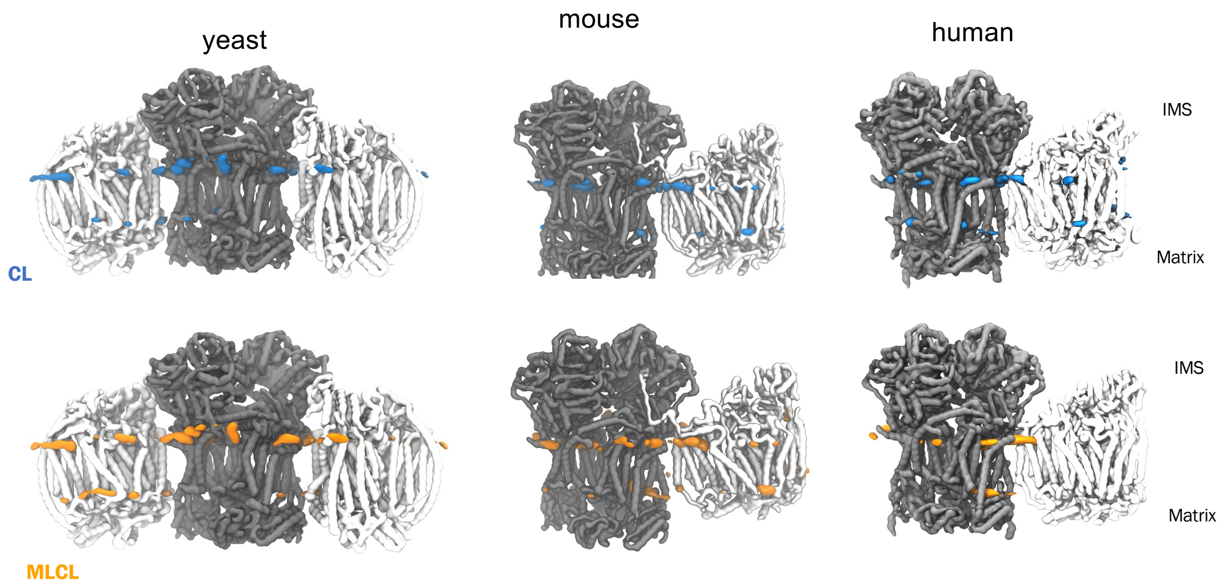

Figure S1. CL (top row) and MLCL (second row) headgroup density from yeast, mouse and human $CIII_2CIV_{1\text{-}2}$ CG MD simulations. CIII backbone is shown in grey and CIV in white, with CL headgroup densities in blue and MLCL densities in orange.

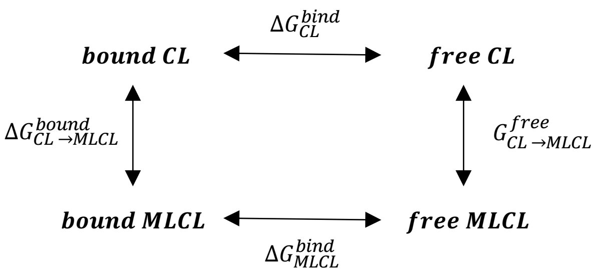

$$\Delta\Delta G = \Delta G_{CL}^{bind} - \Delta G_{MLCL}^{bind}$$
$$= \Delta G_{CL\rightarrow MLCL}^{bound} - \Delta G_{CL\rightarrow MLCL}^{free}$$

Figure S2. Thermodynamic cycle used for the free energy perturbation simulations.

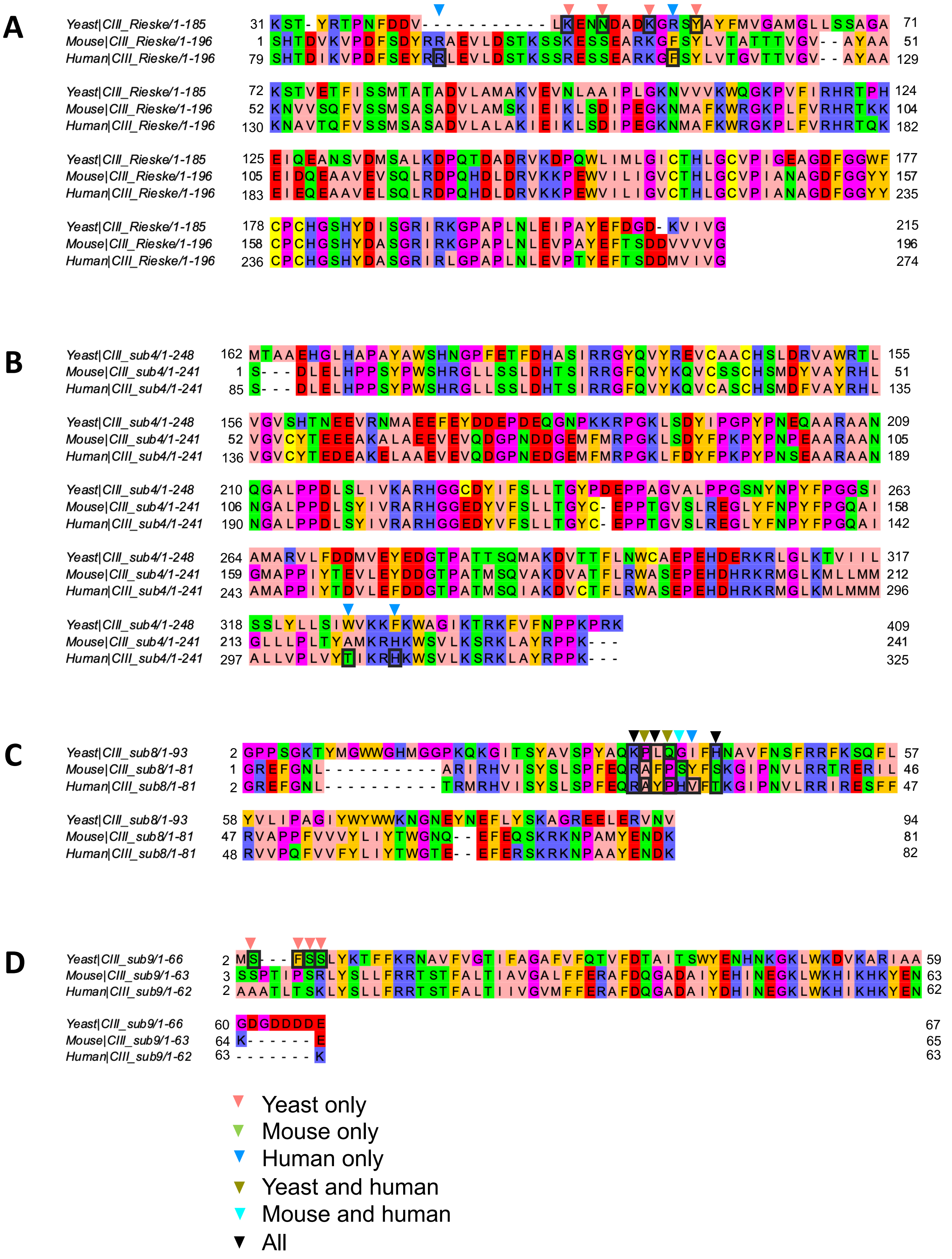

Figure S3. Sequence alignment of the yeast, mouse and human subunits of Complex III that were engaged in MLCL or CL interactions during simulations, showing **A)** the Rieske subunit; **B)** subunit 4; **C)** subunit 8; and **D)** subunit 9. Residues identified as being in the interface interaction site with occupancy > 40 % (see also Table 2) are indicated on the sequence alignment by a black outline and arrow (arrow colour shows the species involved in the interaction)

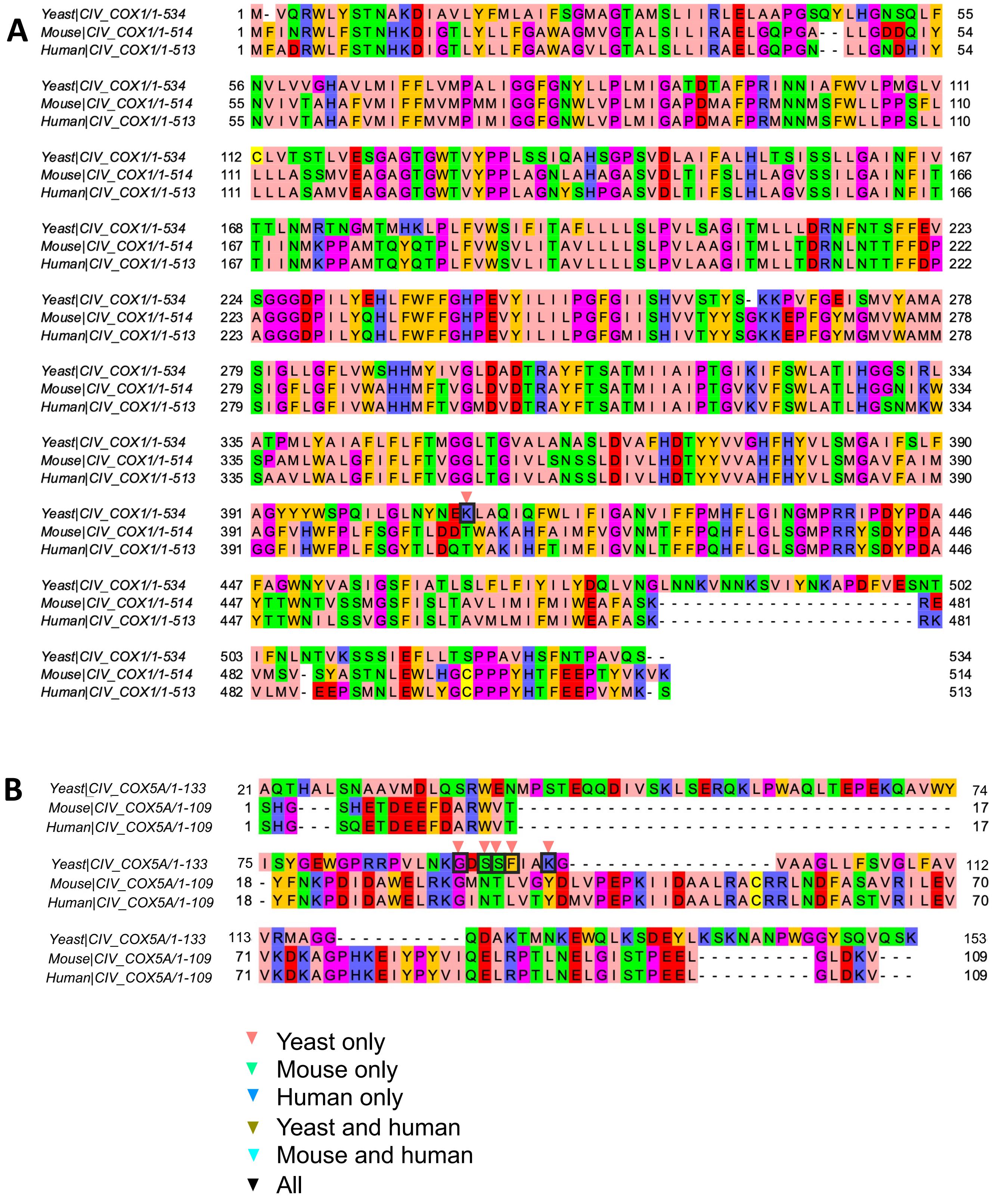

Figure S4. Sequence alignment of the yeast, mouse and human **A)** COX1; **B)** COX5A subunits of CIV. Together with Figure S5, these comprise all the subunits of CIV that were engaged in MLCL or CL interactions during simulations. Residues identified as being in the interface interaction site with occupancy > 40 % (see also Table 2) are indicated on the sequence alignment by a black outline and arrow (arrow colour shows the species involved in the interaction)

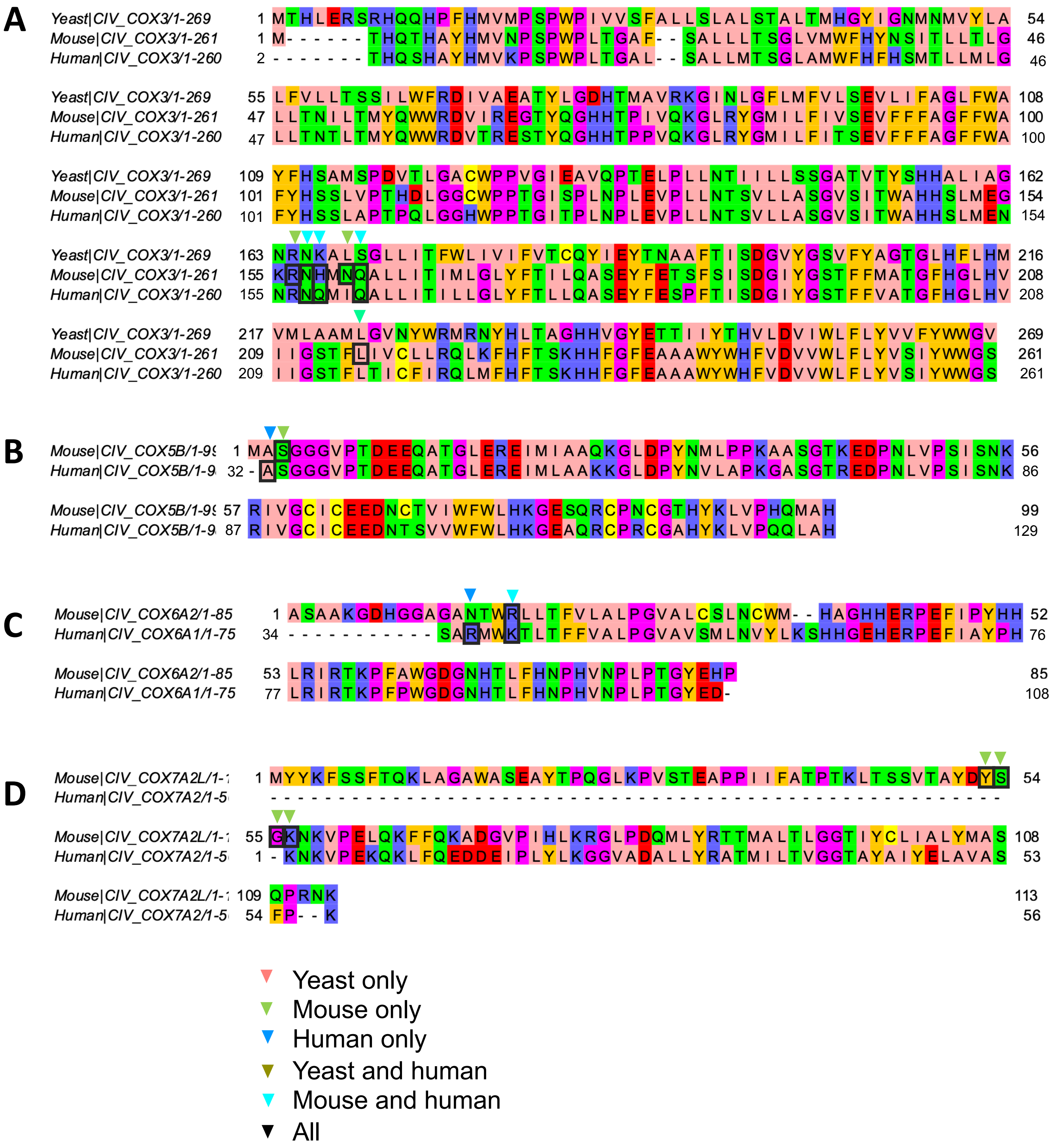

Figure S5. Sequence alignment of the yeast *(S. crevisiae),* mouse and human **A)** COX3; and mouse and human **B)** COX5B; **C)** COX6A; and **D)** COX7A2 subunits of CIV. Note that yeast CIV does not contain subunits COX5B, COOX6A, nor COX7A2. Together with Figure S4, these comprise all the subunits of CIV that were engaged in MLCL or CL interactions during simulations. Residues identified as being in the interface interaction site with occupancy > 40 % (see also Table 2) are indicated on the sequence alignment by a black outline and arrow (arrow colour shows the species involved in the interaction)

Table S1. Residence time and occupancy for each interface site overall, and individually for each residue within the site, as listed in Table 2

Lipid	Complex and Subunit		Residue	Res. time (μs)	Occupancy (%)
Yeast					
CL (Site 1)			Overall:	11.14	98.46
	CIII	Rieske	K44	8.87	48.57
			K51	1.91	73.51
		Sub 8	K35	3.89	42.83
		Sub 9	S2	0.69	84.99
			F3	0.59	43.05
			S4	0.63	53.20
			S5	0.70	66.89
	CIV	COX5A	S93	7.48	45.48
			F94	3.92	43.49
			K97	3.89	40.40
CL (Site 2)			Overall:	12.05	100.00
	CIII	Rieske	K44	3.78	47.68
			K51	1.25	74.17
			W55	3.91	49.23
		Sub 8	K35	9.80	81.02
			P36	8.55	55.85
			L37	9.06	47.02
		Sub 9	S2	0.95	93.38
			S4	0.81	52.32
			S5	2.17	66.89
	CIV	COX5A	S93	7.71	49.45
			K97	9.27	71.97
MLCL (Site 1)			Overall:	10.21	93.84
	CIII	Rieske	K44	4.36	76.91
			N46	2.03	41.96
			K51	1.66	68.77
		Sub 8	K35	6.12	63.95
	CIV	COX1	K408	2.36	46.13
		COX5A	G90	1.98	41.71
			F94	4.08	59.98
			K97	6.49	61.52
MLCL (Site 2)			Overall:	15.10	100.00
	CIII	Rieske	K44	14.06	73.82
			N46	14.36	49.40
			K51	1.24	71.42
			Y55	6.64	61.75
		Sub 8	K35	13.30	94.28
			P36	3.94	48.11
			L37	3.68	46.74
			Q38	8.94	45.42
			H42	1.60	47.71
	CIV	COX5A	G90	6.90	50.33
			S92	3.69	48.17
			S93	7.06	71.20
			F94	7.07	72.09
			K97	12.81	87.01

Lipid	Complex and Subunit		Residue	Res. time (μs)	Occupancy (%)
Mouse					
CL			Overall:	15.00	100.00
	CIII	Sub 8	R24	8.18	71.74
			F26	6.36	62.03
			S28	6.23	65.56
			S31	2.71	56.95
	CIV	COX3	R156	2.21	48.79
			N157	15.00	89.18
			H158	8.07	66.67
			Q161	7.71	60.93
			L223	14.43	59.16
		COX5B	S3	8.19	71.08
		COX6A2	R17	6.55	56.51
		COX7A2L	S54	2.59	75.94
			G55	2.71	62.69
			K56	1.97	96.47
MLCL			Overall:	8.48	99.12
	CIII	Sub 8	R24	7.11	41.72
			S31	0.56	50.33
	CIV	COX3	R156	15.00	76.60
			N157	9.02	83.22
			H158	7.17	42.61
			N160	15.00	58.50
			L223	15.00	81.02
		COX5B	S3	7.10	41.50
		COX7A2L	Y53	2.29	9.27
			S54	15.00	90.51
			G55	15.00	80.57
			K56	10.53	96.69
Human					
CL			Overall:	15.00	100.00
	CIII	Rieske	R93	15.00	87.20
			F113	15.00	77.70
		Sub 4	T305	15.00	41.72
			H309	15.00	93.38
		Sub 8	R25	15.00	97.79
			A26	15.00	65.12
			Y27	15.00	63.36
			P28	2.29	49.67
			H29	3.13	56.73
			V30	1.71	43.27
			T32	2.37	59.16
	CIV	COX3	N157	3.02	75.94
			Q158	15.00	83.44
		COX5B	A32	15.00	83.22
		COX6A1	R36	15.00	81.24
			K39	15.00	95.81
MLCL			Overall:	15.00	100.00
	CIII	Rieske	R93	15.00	90.95
			F113	15.00	79.03
		Sub 4	T305	15.00	43.27
			H309	15.00	92.27
		Sub 8	R25	15.00	98.23
			A26	15.00	52.76
			Y27	14.55	60.71
			P28	3.46	57.62
			H29	15.00	69.32
			V30	1.44	47.02
			T32	2.04	65.56
	CIV	COX3	N157	3.82	75.28
			Q158	15.00	80.57
			Q161	2.35	44.15
		COX5B	A32	15.00	89.40
		COX6A1	R36	15.00	87.20
			K39	15.00	95.14
